# Negative outcomes associated with tyrosine kinase inhibitors during management of gastrointestinal stromal tumors: examination of data from the FDA adverse event reporting system

**DOI:** 10.3389/fonc.2025.1608451

**Published:** 2025-08-29

**Authors:** Yuxuan Ma, Ahui Fan, Yuhao Wang, Shenhui Xu, Xin Liu, Jianjun Yang

**Affiliations:** ^1^ Department of Digestive Surgery, Xijing Hospital of Digestive Diseases, Fourth Military Medical University, Xi’an, China; ^2^ State Key Laboratory of Holistic Integrative Management of Gastrointestinal Cancers and National Clinical Research Center for Digestive Diseases, Xijing Hospital of Digestive Diseases, Fourth Military Medical University, Xi’an, China

**Keywords:** gastrointestinal stromal tumors, tyrosine kinase inhibitors, adverse effects, FDA adverse event reporting system database, pharmacovigilance

## Abstract

**Background:**

Since the approval of Tyrosine kinase inhibitors (TKIs) in gastrointestinal stromal tumors (GISTs), the survival of patients with metastatic GISTs have been remarkably improved. But clinically, the adverse effects (AEs) are the major barrier to the long-term and standardized treatment and less reported.

**Methods:**

The data was acquired from FDA Adverse Event Reporting System (FAERS) database from 2006 to 2024. TKIs were selected based on the clinical guidelines for the treatment of GISTs. AEs induced by different TKIs were analyzed using calculating reporting odds ratios (ROR), the proportional reporting ratio (PRR), the Bayesian confidence propagation neural network (BCPNN), and the multi-item gamma-Poisson shrinker (MGPS) to compare the character of safety signals of different TKIs.

**Results:**

Disorders affecting skin and subcutaneous tissue, and the circulatory system emerged as the most common adverse events elicited by the majority of tyrosine kinase inhibitors. Meanwhile, some AEs only were observed in certain TKI. Endocrine disorders have higher risk only during the treatment with sunitinib, while avapritinib displayed unique AEs associated with nervous system disorders. Additionally, several significant signals were found on the preferred term (PT) level, including brain fog with avapritinib (ROR = 27.72), pemphigus (ROR = 30.90) with imatinib, nerve injury (ROR = 25.02) with ripretinib, tough blistering (ROR = 54.96) with sunitinib.

**Conclusion:**

Our comprehensive pharmacovigilance analysis identified distinct adverse event profiles and significant drug-specific safety signals among TKIs used in GIST treatment. These findings enhance the characterization of TKI safety, revealing previously unreported or strongly associated signals and highlighting differences between agents. This evidence contributes to a better understanding of TKI-associated risks in clinical practice.

## 1 Introduction

Gastrointestinal stromal tumors (GISTs) are the most frequent sarcoma in alimentary tract, exhibiting an increasing incidence worldwide, particularly among Asian and Pacific Islanders or black people (1). Mechanically, the mutation on tyrosine kinase receptors, c-KIT and PDGFR, are generally considered to drive the most of GISTs development and progression (2). Since imatinib, a kind of tyrosine kinase inhibitor (TKI), was approved for use in GISTs therapy in 2002 (3), there has been a remarkable enhancement in the outlook for individuals afflicted with metastatic GISTs. However, the AEs of TKIs are the major barrier to the long-term and standardized treatment for patients.

Globally, TKIs have received clinical approval for initial therapy in individuals with metastatic GISTs. After Imatinib approved for use in GISTs in 2002, sunitinib was approved as second-line therapy in 2006, avapritinib and ripretinib were both approved in 2020. With its widespread application, the AEs especially some rare adverse events, have gradually emerged hindering clinical applications (4). Although there have been several clinical trials reporting some AEs, a systematic real-world analysis is still scarce. Additionally, the NCCN guideline (2022) just illustrated the AEs of imatinib and sunitinib but lack the description of AEs in other TKIs (5). Moreover, the time onset and demographic features remain unclear. Consequently, it is imperative that we clearly delineate the negative impact associated with the use of TKIs in therapy to ensure their secure utilization in medical practice.

The FDA Adverse Event Reporting System database (FAERS) is a significant worldwide database for monitoring pharmaceuticals after they are released on the market, acts as a tool for gathering data on unexpected AEs via voluntary submissions, thus facilitating the prompt detection of medication-related safety issues within broad demographic settings (6).This study primarily aimed to systematically identify and compare the AE spectra associated with four distinct tyrosine kinase inhibitors, namely imatinib, sunitinib, avapritinib and ripretinib that approved for GISTs treatment. We sought to detect potential drug-specific safety signals and previously unreported or unexpected AE signals strongly linked to individual TKIs. Finally, the study aimed to provide a comprehensive, real-world evidence-based comparison of the AE profiles across these agents.

## 2 Methods

### 2.1 Data source and collection

We collected data from the FAERS database published between January 2006 and August 2024. The data of the FAERS database encompass information such as the gender, age, medication duration, occurrence time, dosage, causality, and prognosis of the individuals reporting AEs. In cases of multiple reports related to the same event, a protocol for duplicate data removal was executed in accordance with FDA guidelines.

### 2.2 Data processing

From “January 2006” to “August 2024”, after limiting the indications to GISTs, the cases in FAERS reports where “imatinib”, “sunitinib”, “avaprtinib” and “ripretinib” were listed as suspects. After removing duplicates (with the same ID number), we obtained a total of 12,353 suspected AEs caused by these four TKIs during the treatment of GISTs, the deduplication process was performed using RStudio 4.2.3. Two researchers classified TKIs-related AEs using preferred term (PT) and system organ classification (SOC). Moreover, The MedDRA (Medical Dictionary for Regulatory Activities) dictionary (Version 27.1) was used to classify confirmed PTs into specific SMQs. SMQ is a predefined set of MedDRA terms related to specific medical conditions or safety concerns. Subsequently, the disproportionality analysis was conducted on the level of PT, SOC and SMQ to determine the association between TKIs and AEs.

### 2.3 Statistical analysis

Patient demographics with GISTs undergoing TKIs treatment were illustrated through averages and variability measures for continuous data, and counts and proportions for categorical data. To discern the correlations between pharmaceuticals and negative reactions, methodologies such as the relative odds ratio (ROR), the proportional reporting ratio (PRR), the Bayesian confidence propagation neural network (BCPNN), and the multi-item gamma-Poisson shrinker (MGPS) were employed (7, 8). The meaning of each If any one of the previously mentioned four benchmarks were met, the AE was considered to have statistical significance in PT and SOC, as noted in **Table 1**, the meanings of each letter in these formulas are shown in the form of a Two-by-two contingency table (**Table 2**). For SMQ, we used a single ROR to determine the significance of the association between AEs and TKIs, the correspondence table between SMQ and PT is presented in the **Supplementary Table**.

**Table 1:** The formulas and criteria for the four algorithms (a: The co-occurrence report count of the target drug and the target AEs in GISTs treatment, b: The report count of the target drug and other AEs in GISTs treatment, c: The report count of non-target drugs and the target AE in GISTs treatment, d: The report count of non-target drugs and other AEs in GISTs treatment).

Method	Calculation	Criteria
ROR	ROR=ad/bc 95%CI=eln_(ROR)_ ± 1.96(1/a +1/b + 1/c + 1/d)^0.5^	95%CI > 1, N ≥ 3
PRR	PRR=a(c+d)/c(a+b) χ^2^=[(ad-bc)^2^](a+b+c+d)/[(a+b)(c+d)(a+c)(b+d)]	PRR ≥2, χ^2^ ≥4, N ≥ 3
BCPNN	IC = log_2a_(a + b + c + d)(a + c)(a + b) 95% CI = eln_(IC)_ ± 1.96(1/a+1/b+1/c+1/d)^0.5^	IC_0.25_ > 0
MGPS	EBGM = a(a + b + c + d)/(a + c)/(a + b) 95% CI = eln_(EBGM)_ ± 1.96(1/a+1/b+1/c+1/d)^0.5^	EBGM_0.5_ > 2,N > 0

**Table 2:** Two-by-two contingency table for disproportionality analyses.

	Adverse events of interest in GIST treatment	All other adverse events of interest in GIST treatment	Total
Drug of interest	a	b	a+b
All other drugs of interest	c	d	c+d
Total	a+c	b+d	a+b+c+d

## 3 Results

### 3.1 Characteristics of patients

**Figure 1:**
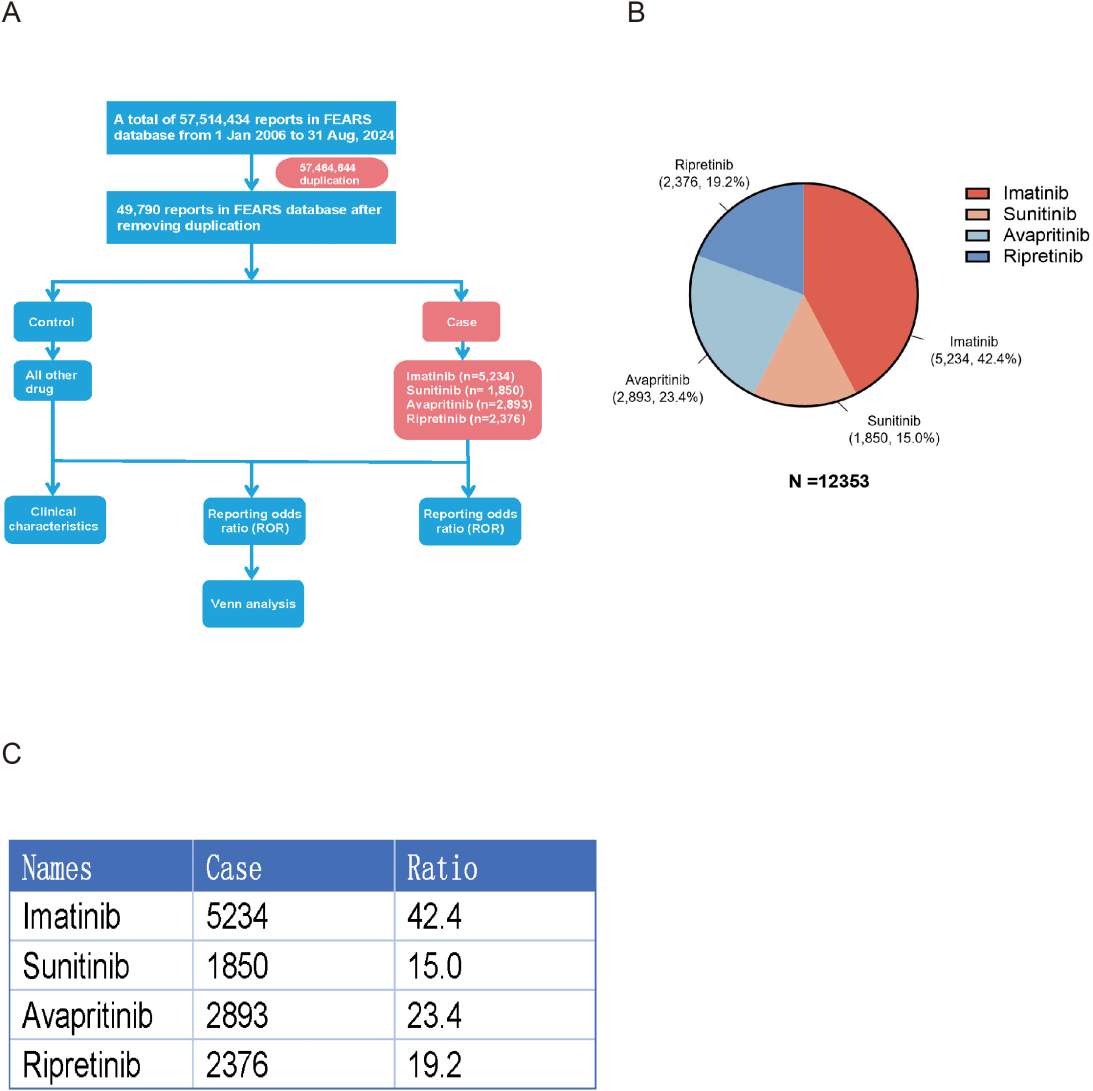
**(A)** Flowchart of study design. After duplication, 5,234 cases led by imatinib, 1,850 cases led by sunitinib, 2,893 cases led by avapritinib and 2,376 cases led by ripretinib were involved in this study. **(B)** Pi-chart representing the total number of cases and its distribution of individual TKIs. **(C)** Table representing the total number of cases and its distribution of individual TKIs.

As shown, a total of 12,353 reports related with AEs of TKIs in GISTs treatment were reported in the FAERS database from January 2006 to August 2024. The reports numbered 5,234 (42.37%) for imatinib, 1,850 (14.98%) for sunitinib, 2,893 (23.42%) for avapritinib and 2,376 (19.23%) for ripretinib (**Figure 1**). **Table 3** encapsulates a summarization of the demographic features documented in the pharmacological reports.

**Table 3:** Clinical characteristics of TKIs in GISTs treatment reported in the FAERS database.

Characteristics				
	Imatinib	Sunitinib	Avapritinib	Ripretinib
Gender				
Female	2103 (40.2)	766 (41.4)	1279 (44.2)	1035 (43.6)
Male	2771 (53.0)	968 (52.3)	1575 (54.4)	1324 (55.7)
Unknown	357 (6.8)	116 (6.3)	39 (1.3)	17 (0.7)
Age				
<18	23 (0.4)	4 (0.2)	15 (0.5)	1 (0.0)
18-64	1511 (28.9)	794 (42.9)	697 (24.1)	424 (17.9)
>65	1452 (27.8)	829 (44.8)	1102 (38.1)	590 (24.8)
Unknown	2245 (42.9)	223 (12.1)	1079 (37.3)	1361 (57.3)
Weight				
<50 kg	65 (1.2)	65 (3.5)	2 (0.1)	22 (0.9)
>100 kg	60 (1.1)	36 (1.9)	13 (0.4)	11 (0.5)
50∼100 kg	535 (10.2)	458 (24.8)	63 (2.2)	178 (7.5)
Unknown	4571 (87.5)	1291 (69.8)	2815 (97.3)	2165 (91.1)
Report countries				
US	1107 (21.2)	797 (43.1)	2626 (90.8)	2177 (91.6)
Non-US	2935 (55.1)	1051 (56.8)	267 (9.2)	195 (8.2)
Unknown	1242 (23.7)	2 (0.1)	1 (0)	4 (0.2)
Reporter role				
Health profession	3133 (59.9)	998 (53.5)	239 (8.3)	914 (38.5)
Non-Health profession	1935 (37.0)	805 (53.4)	2633 (91.0)	1460 (61.4)
Unknown	163 (3.1)	57 (3.1)	21 (0.7)	2 (0.1)
Outcome				
Death/Life-threatening	2421 (46.3)	609 (32.9)	113 (3.9)	281 (11.8)
Disability	39 (0.7)	8 (0.4)	4 (0.2)	/
Hospitalization	648 (12.4)	456 (24.6)	274 (9.5)	446 (18.8)
Other serious	1574 (30.1)	377 (20.4)	421 (14.5)	260 (10.9)
Required intervention	3 (0.1)	3 (0.2)	/	/
Congenital Anomaly	2 (0.0)	/	/	/
Unknown	544 (10.4)	397 (21.5)	2081 (71.9)	1389 (58.5)

**Figure 2:**
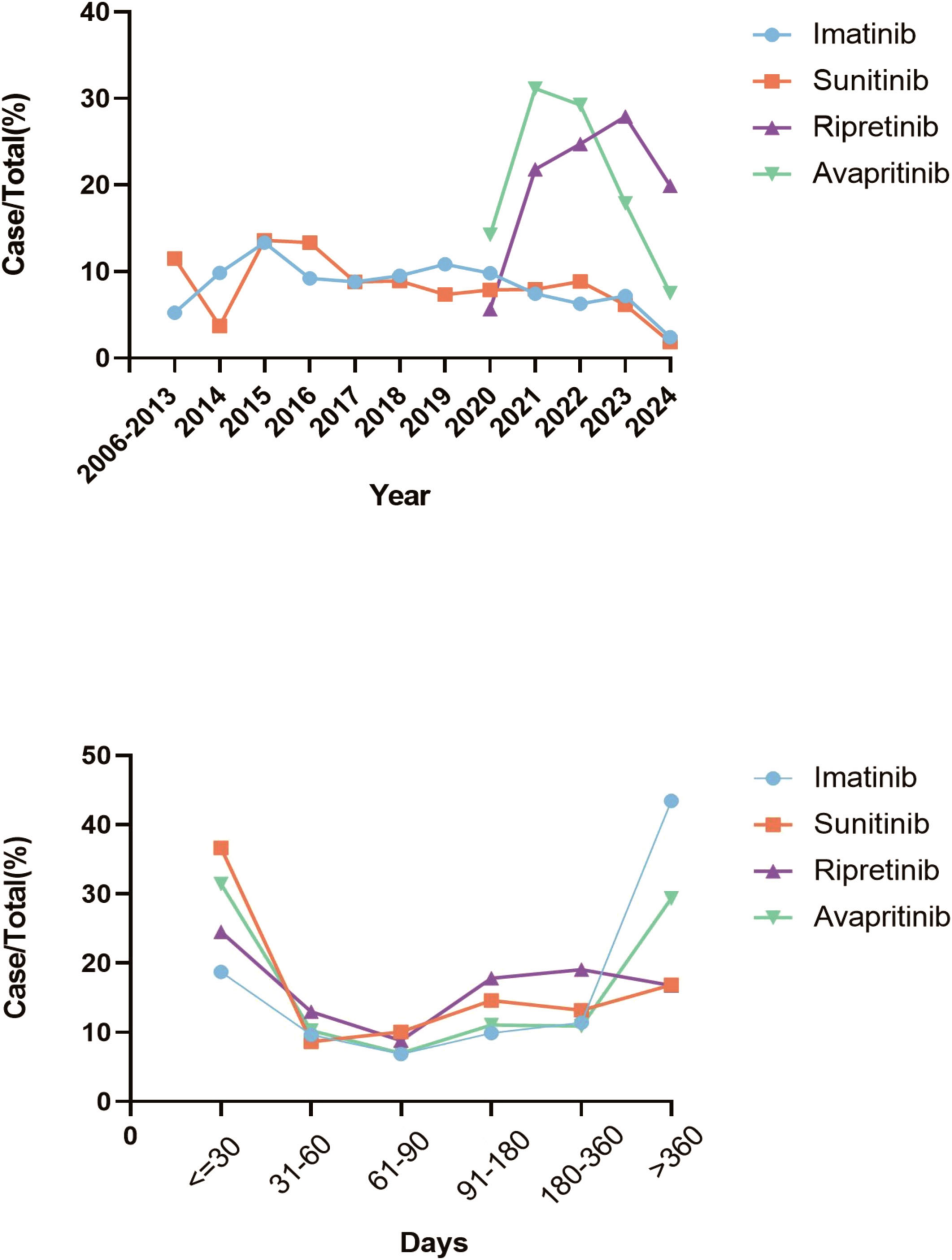
Trends in AEs for all four TKIs. **(A)** The trends of AEs for TKIs from 2006 to 2024 indicated that the number of reports for ripretinib and avapritinib increased during the first 1–3 years after launch, then declined in subsequent years. **(B)** The time-to-onset analysis of TKIs indicated that most AEs occurred in the first month of treatment and one year later.

In terms of gender-related variations, male patients experienced more frequent AEs than female patients during TKIs treatment. Among patients under 18 years old, AE reports were the lowest. No significant age-related differences in AE reports were observed for imatinib and sunitinib users. However, patients over 65 years old experienced more AEs than those aged 18–65 when using avapritinib and ripretinib. The reports of imatinib and sunitinib were mostly originated form health-professions, accounting for 59.9% and 53.5% respectively, compared with 8.3% in avapritinib and 38.5% in ripretinib, and these reports from non-health profession were mainly from US, China and Japan. Additionally, death was the main recorded outcome for imatinib (n = 2,421) and sunitinib (n = 609), while hospitalization for avapritinib (n = 274) and ripretinib (n = 446). The time distribution analysis of post-marketing AE reports shows that ripretinib and avapritinib had an increase in reports during the first 1–3 years after launch, followed by a decline in later years. In contrast, imatinib and sunitinib had very few reports during their early post-marketing period, making it difficult to assess initial trends. To better understand their long-term reporting patterns, charts for these two drugs display annual report counts for eight years or more after market entry.– Notably, AEs mostly occurred within the first month of therapy and again after one year (**Figure 2**).

### 3.2 AEs distribution analyzed on the level of system organ classification and the preferred term

**Figure 3:**
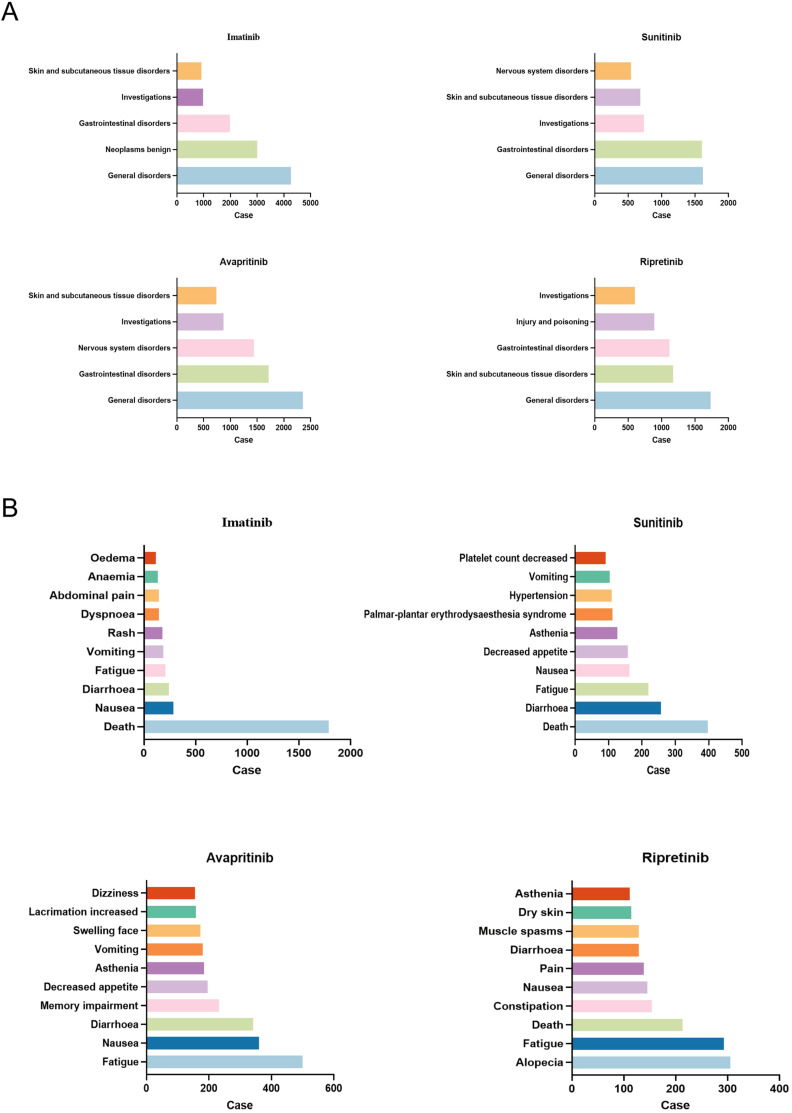
AEs most frequent for all four TKIs. **(A)** The top five AEs related to certain type of TKIs at SOC level were shown. General disorders was the most frequent SOC in all TKIs. **(B)** The top ten AEs related to certain type of TKIs at PT level were shown. Death was the most frequent PT in imatinib and sunitinib, while fatigue was the most common PT in avapritinib, alopecia was most common in ripretinib.

Subsequently, we analyzed the hierarchy of AEs according to the System Organ Class (SOC) and PT measurements across the quartet of pharmaceuticals, with **Figure 3** displaying the leading 10 AEs for PT and the quintet of AEs for SOC. The original data contains all PT led by TKIs was shown in **Supplementary 1**. Within the SOC category, general disorders, skin and subcutaneous tissue disorders, gastrointestinal disorders and investigation, which means all results obtained through medical examinations are present in all four drugs, with general disorders ranking as the predominant AEs.

In the level of PT, diarrhea and fatigue were the most common AEs. It was noteworthy that each drug exhibited idiosyncratic AEs. For example, nervous system specific disorders such as dizziness and memory impairment were unique to avapritinib, hypertension and platelet count decreased were specific to sunitinib, anemia and edema were more common in imatinib than the others, alopecia was unique to ripretinib (**Figures 3A, B**).

### 3.3 Disproportionality analysis of SOC

**Figure 4:**
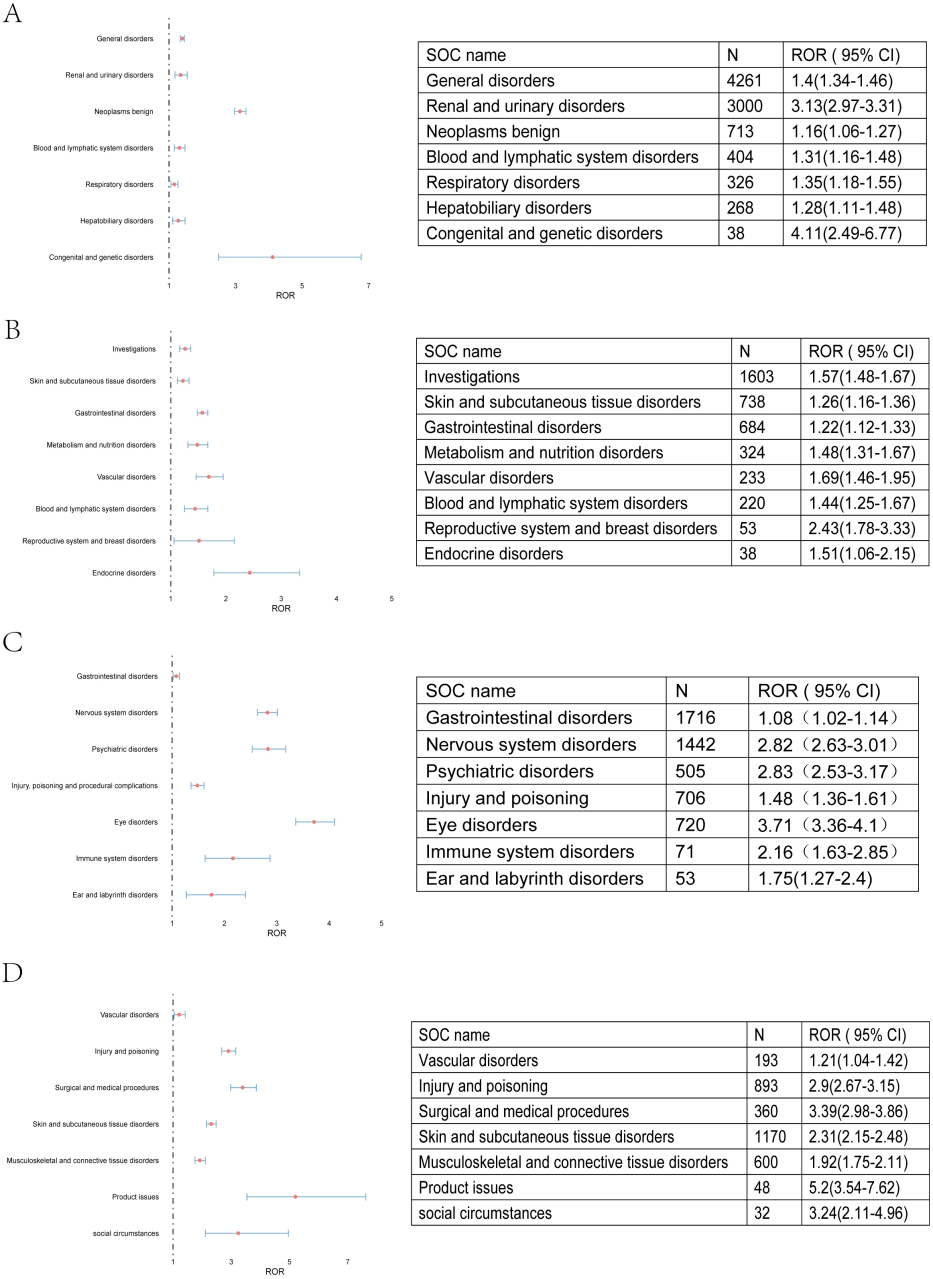
AEs related to all four TKIs at SOC level. **(A)** AEs related to imatinib were shown. Congenital and genetic disorders has the highest ROR value. **(B)** AEs related to sunitinib were shown. Reproductive system and breast disorders has the highest ROR value. **(C)** AEs related to avapritinib were shown. Eye disorders has the highest ROR value. **(D)** AEs related to ripretinib were shown. Product issues has the highest ROR value.

**Figure 5:**
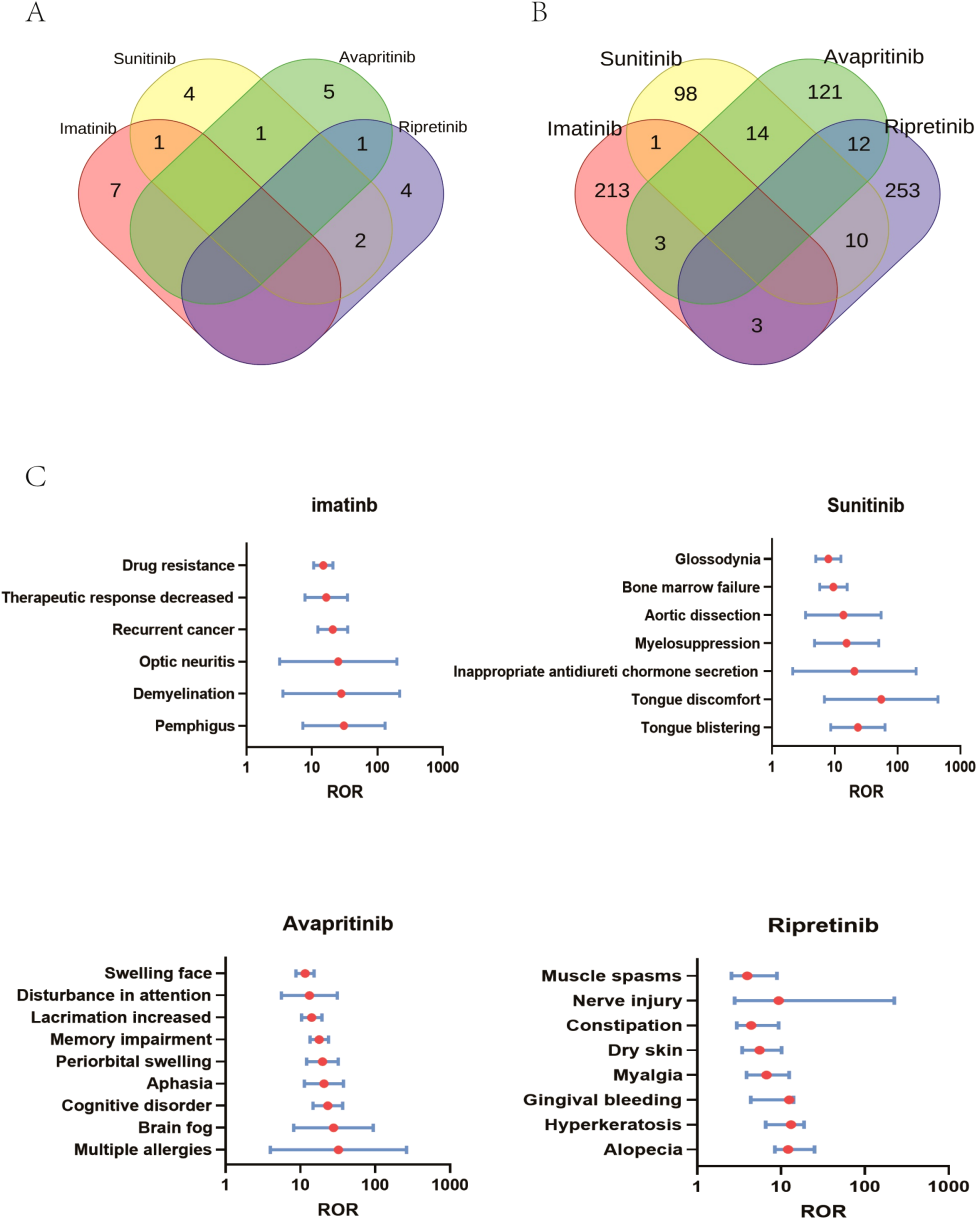
SOC specialized in certain type of TKIs. **(A)** AEs specialized in TKIs on SOC level: At the SOC level, 8 AEs were associated with imatinib and sunitinib, while 7 AEs were linked to the other two drugs. Specifically, 7 SOCs were unique to imatinib, 4 to sunitinib, 5 to avapritinib, and 4 to ripretinib. **(B)** AEs specialized in TKIs on PT level: At the PT level, 220 AEs were associated with imatinib, 123 AEs were associated with sunitinib, 150 AEs were associated with avapritinib, 278 AEs were associated with ripretinib. Specifically, 213 AEs were unique to imatinib, 98 to sunitinib, 121 to avapritinib, and 253 to ripretinib. **(C)** Comparison of significant AEs at the PT level among four TKIs showed PTs with ROR values over 10. 6 PTs, including drug resistance, were significantly linked to imatinib, 7, including tongue discomfort, to sunitinib, 9 to avapritinib and 8 to ripretinib.

The signal of strengths of imatinib, sunitinib, avapritinib and ripretinib across different SOC were analyzed using the criteria for the 4 algorithms (ROR, PRR, BCPNN, MGPS). The signal of SOC was considered significant when at least one algorithm was fulfilled. ROR was used in the comparison of SOC in these four TKIs. The SOCs that were considered as close correlated with these TKIs by the disproportionality analysis were shown in color code. Imatinib were shown in “red”, the SOC related to sunitinib in “yellow”, SOC related to avapritinib and ripretinib by “green” and “purple”, respectively. The counts of SOCs related to imatinib, sunitinib, avapritinib and ripretinib were 8, 8, 7, 7 respectively. Subsequently, the Venn analysis was performed. The analysis indicated that there were 7 SOC specific to imatinib, with congenital and genetic disorders had the highest ROR value. 4 SOC were specific to sunitinib and endocrine disorders had the highest ROR value. 5 SOC were specific to avapritinib and eye disorders had the highest ROR value. 4 SOC were specific to ripretinib, surgical and medical procedures had the highest ROR value. There were still some SOC observed in the group of two or more TKIs, including injury and poisoning, skin and tissue disorders, vascular disorders and gastrointestinal disorders (**Figures 4**, **5A**). Although some SOCs were observed in more than one TKIs, the PT distribution differed. Diarrhea was the most commonly reported gastrointestinal adverse event, particularly linked to sunitinib and avapritinib. Among gastrointestinal conditions, sunitinib showed stronger associations with intestinal obstruction (ROR, enteritis and ischemic colon damage. In contrast, constipation was more strongly linked to ripretinib.

### 3.4 Comparison of significant AEs across imatinib, sunitinib, avapritinib and ripretinib

Similar to the analysis of SOCs, PTs were presented in the same way, with PTs related to imatinib shown in “red”, PT related to sunitinib in “yellow”, and PT related to avapritinib and ripretinib in “green” and “purple”, respectively. Venn analysis indicated that 213 AEs specialized to imatinib was detected, and the number of AEs specialized to sunitinib, avapritinib and ripretinib were 98,121 and 253 respectively (**Figure 5A**). The different AEs related to certain type of TKIs was described in **Figure 5**. Imatinib was correlated with skin and subcutaneous tissue disorders, including pemphigus (ROR (95% CI) = 30.9 (7.26-131.41), N = 22), demyelination (ROR (95% CI) = 28.07 (3.59-219.3), N = 10) and optic neuritis (ROR (95% CI) = 25.26 (3.2-199.41), N = 9). Sunitinib was correlated with tongue discomfort (ROR (95% CI) = 23.28 (8.62-63.39), N = 17), and vascular disorders such as myelosuppression (ROR (95% CI) = 15.46 (4.76-50.21), N = 9). Injury and poisoning was highly correlated with avapritinib and riprertinib. Multiple allergies was connected with avapritinib (ROR (95% CI) = 32.31 (3.97-262.64), N = 7) and nerve injury was connected with ripretinib (ROR (95% CI) = 25.02 (2.8-223.93), N = 4). Nervous system disorders was specifically connected with avapritinib, the highest signals among which were cognitive disorders (ROR (95% CI) = 23.28 (14.72-36.81), N = 110) and aphasia (ROR (95% CI) = 20.68 (11.33-33.75), N = 58) (**Figures 5B, C**).

### 3.5 Disproportionality analysis of SMQ

**Figure 6:**
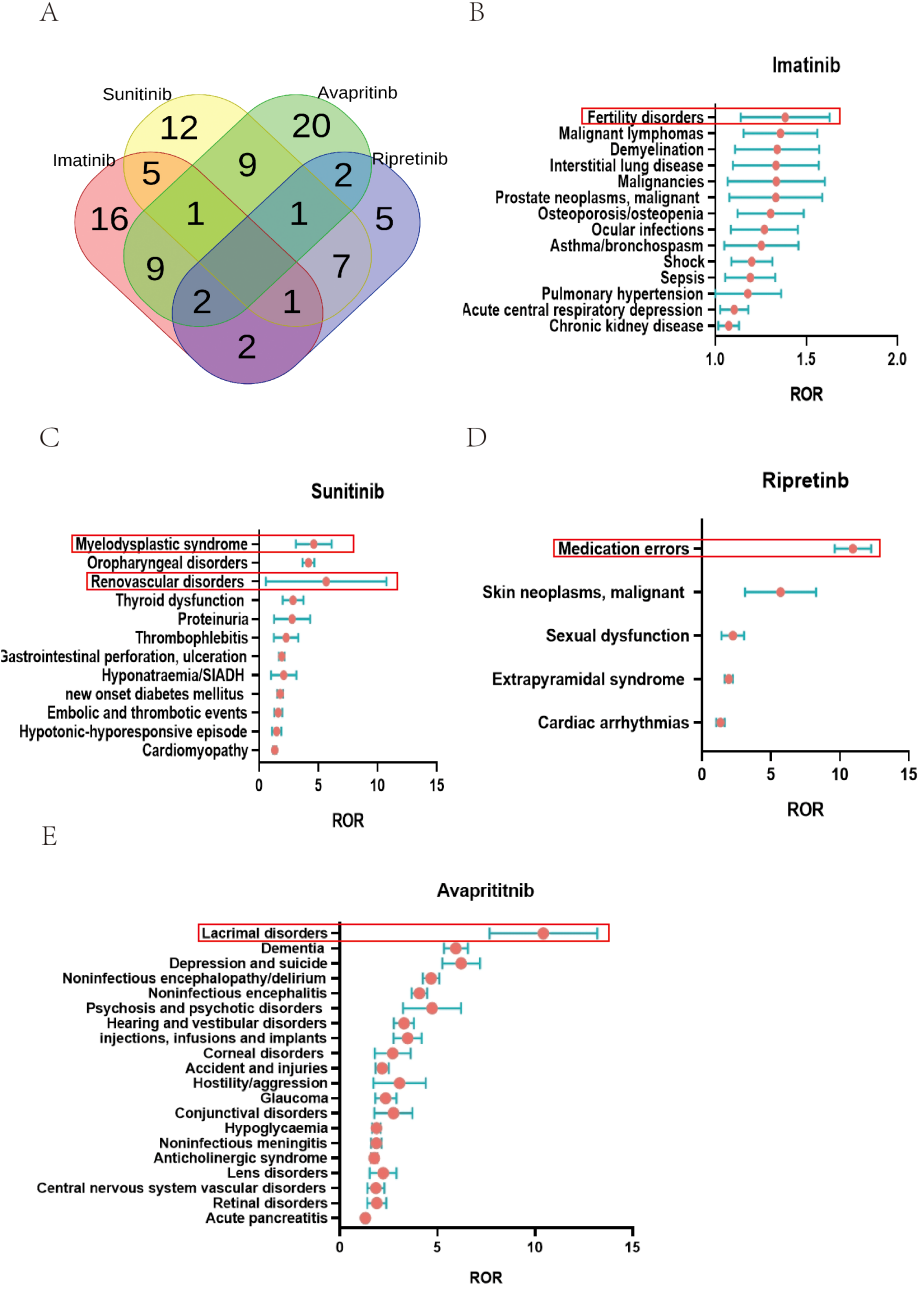
SMQ specialized in certain type of TKIs. **(A)** AEs specialized in TKIs on SMQ level: At the SMQ level, Imatinib, sunitinib, avapritinib, and ripretinib were associated with 36, 36, 44, and 20 SMQs, respectively. Imatinib had 16 specific SMQs, sunitinib had 12 specific SMQs, avapritinib had 20 specific SMQs, ripretinib had 5 specific SMQs. **(B)** SMQs related to imatinib were shown, with reproductive disorders showing the highest ROR value. **(C)** SMQs related to sunitinib were shown, with tumor lysis syndrome showing the highest ROR value. **(D)** SMQs related to ripretinib were shown, with medication errors showing the highest ROR value. **(E)** SMQs related to avapritinib were shown, with lacrimal disorders showing the highest ROR value.

The ROR method was used to analyze the signal strength of imatinib, sunitinib, avapritinib, and ripretinib across different AEs (SMQs). Color codes were applied to indicate SMQs closely associated with each TKI. Red for imatinib, yellow for sunitinib, green for avapritinib, and purple for ripretinib. The number of related SMQs for imatinib, sunitinib, avapritinib, and ripretinib was 36, 36, 44, and 20, respectively. Venn diagram analysis revealed that imatinib had 16 specific SMQs, with Fertility disorders showing the highest ROR value. Sunitinib had 12 specific SMQs, led by Tumour lysis syndrome. Avapritinib had 20 specific SMQs, dominated by Lacrimal disorders. Ripretinib had 5 specific SMQs, with Medication errors having the highest ROR (**Figure 6A**, **Supplementary Table 4**). Some SMQs, including Opportunistic infections, Peripheral neuropathy, Osteonecrosis, and Drug reaction with eosinophilia and systemic symptoms syndrome, were observed in combinations of two or more TKIs. The unique SMQs of the four drugs are presented in forest plots in **Figures 6B–E**. Among the unique SMQs of imatinib, Fertility disorders had the highest ROR value (ROR (95% CI) = 1.36 (1.15 - 1.63), N = 184). Among the unique SMQs of sunitinib, Renovascular disorders had the highest ROR value (ROR(95% CI) = 4.45 (3.19 - 6.19), N = 58). Among the unique SMQs of avapritinib, Lacrimal disorders had the highest ROR value (ROR(95% CI) = 10.18 (7.79 - 13.30), N = 172). Among the unique SMQs of ripretinib, Medication errors had the highest ROR value (ROR(95% CI) = 10.89 (9.65 - 12.30), N = 711).

## 4 Discussion

Drawing on information from the FAERS, our in-depth examination focused on the pattern of AEs related to the quartet of FDA-sanctioned TKIs employed in the management of GISTs. GISTs are typically caused by mutations in KIT or PDGFR genes, leading to inappropriate tyrosine kinase activation and promoting tumor development (9). Thus, drugs targeting KIT, PDGFR and other tyrosine kinases have become the basis of precise targeted therapy for GISTs and have significantly improved the prognosis (10). However, clinical observations have shown that the use of TKIs can also cause many AEs. In this study, we reviewed the AEs caused by four TKIs recommended by clinical guidelines for the treatment of GISTs. In addition, we also modeled the clinical characteristics, onset time, prognosis and distribution spectrum of AEs caused by these TKIs in patients.

Our results indicate that the use of the four TKIs in this study may lead to some common AEs, including skin and subcutaneous tissue disorders, gastrointestinal disorders, and neoplasm benign. However, the prevalence and severity of these AEs vary among different TKIs. Previous studies have also pointed out that the most common AEs of TKI drugs in GISTs are fatigue, skin reactions, and gastrointestinal discomfort (11, 12). Our results further confirm this conclusion. The INTRIGUE study revealed the occurrence of Palmar-Plantar Erythrodysesthesia (PPE) among participants, affecting individuals in both the cohort receiving ripretinib (226 patients) and those administered sunitinib (227 patients). Notably, 23 (10%) of the patients being treated with ripretinib developed PPE. In comparison, the incidence of PPE was greater within the sunitinib cohort, with 68 patients affected (13). Research on 204 individuals undergoing avapritinib following an initial unsuccessful intervention for GISTs revealed that the AEs affecting the digestive tract included nausea, diarrhea, and emesis. Out of these, there was a cumulative occurrence of 14 instances (representing 7%) of severe (grade ≥ 3) gastrointestinal AEs (14). In the EORTC 62005 and NASG S0033 phase III trials, 946 patients with unresectable GISTs received treatment at 400 mg or 800 mg per day, with an edema incidence rate of 47%-59% in the 400 mg group and 60%-70% in the 800 mg group (15).

In addition, we also found that certain AEs were specific to certain types of TKIs. To illustrate, the likelihood of experiencing neurological issues (ROR (95% CI) = 2.82 (2.63- 3.01, N = 1442)) and psychiatric conditions (ROR (95% CI) = 2.82 (2.53-3.17, N = 505)) was higher in patients treated with avapritinib, whereas circulatory system complications (ROR (95% CI) = 1.69 (1.46-1.95, N = 223)) and hormonal imbalances (ROR (95% CI) = 2.43 (1.78-3.33, N = 53)) were observed more frequently with sunitinib administration. Regarding digestive system issues across these four TKIs, diarrhea stood out as the most frequently reported AEs. However, it was more closely associated with the use of sunitinib and avapritinib rather than the other two. This result validates the comparison of ripretinib and sunitinib in INTRIGUE and provides more information for comparison with other TKIs. In the panorama of gastrointestinal conditions, sunitinib exhibited a more pronounced indication compared to other medications, with notable effects on intestinal blockage (ROR (95% CI) = 1.74 (1.11-2.72, N = 24)), inflammation of the intestines (ROR (95% CI) = 9.15 (2.05-40.91, N = 4)), and reduced blood flow leading to colonic tissue damage (ROR (95% CI) = 10.30 (10.72-61.64, N = 3)). However, when it came to constipation, ripretinib produced a more significant indication (ROR (95% CI) = 3.61 (2.96-4.41, N = 154)). According to previous clinical trials, the number of patients experiencing grade 3 or 4 AEs was lower in those using ripretinib (n = 592, 41.3%) compared to those using sunitinib (n = 145, 65.6%), which is consistent with our results using FAERS data. As for subcutaneous tissue AEs, serious adverse effects (SAEs) were highly associated with the use of imatinib. The signals of skin necrosis (ROR (95% CI) = 19.65 (2.42-159.69, N = 7)), pyoderma gangrenosum (ROR (95% CI) = 7.02 (1.36-36.16, N = 5)), and pemphigus (ROR (95% CI) = 30.9 (7.26-131.41, N = 22)) were significantly higher in imatinib than in other drugs. PPE had significant signals in both sunitinib and ripretinib, but the signal was higher in sunitinib ((ROR (95% CI) = 3.32 (2.64-4.16, N = 112)) compared to ripretinib ((ROR (95% CI) = 2.11 (1.65-2.69, N = 87)). The signal of avapritinib in subcutaneous tissue disorders was lower, while hair color change had the highest signal in avapritinib ((ROR (95% CI) = 11.7 (8.5-16.1, N = 113)). Since the targets of these TKIs vary, the specific AEs are in need of closer observation.

Our comprehensive imbalance analysis of the Safety Medical Query (SMQ) using the reporting odds ratio (ROR) revealed significant differences in the safety profiles of the four tyrosine kinase inhibitors (TKIs) at the SMQ level. The adverse event patterns of these drugs showed significant heterogeneity, with avapritinib having the broadest safety signal spectrum and ripretinib having a more concentrated safety profile. Venn diagram analysis revealed drug-specific and class-effect safety issues. Notably, imatinib had 16 unique safety signals, with the strongest signal for reproductive disorders, which is consistent with the known gonadotoxicity of BCR-ABL inhibitors. Sunitinib had 12 unique safety signals, with a prominent signal for tumor lysis syndrome, which may reflect its strong anti-angiogenic activity in vascularized tumors. Avapritinib showed a particularly prominent specificity for lacrimal gland disease, which may be related to the role of KIT/PDGFRA inhibition in the lacrimal secretion pathway. Most strikingly, ripretinib showed the highest signal for medication errors, indicating unique challenges in dosing or administration for this novel switch control inhibitor. These findings emphasize that although TKIs share common toxicity mechanisms, their specific kinase inhibition profiles translate into distinct clinical safety profiles. Signal mapping based on SMQ provides a precision toxicology framework for drug-specific risk mitigation strategies.

It is also worth noting that drug interactions may also lead to AEs in the clinical treatment of GISTs with TKIs. As GISTs patients progress from initial imatinib therapy to subsequent ripretinib treatment, the AEs associated with these TKIs can accumulate (16). Previous studies have shown that long-term medication may lead to the accumulation of drug toxicity, including PPE, hypertension, fatigue and hematological toxicity (17). In addition, studies have shown that TKIs should avoid to be used with CYP3A4 inhibitors and inducers to prevent abnormal TKIs blood drug concentrations, thereby increasing liver toxicity or reducing efficacy (18, 19). Moreover, when using immune checkpoint inhibitors, changes in liver function caused by TKIs should also be monitored. Given that the mixture may exacerbate immunological AEs, including colitis, hepatitis, and thyroid abnormalities (20). A recent study on the combination of transcatheter arterial chemoembolization and TKIs has shown that although this combination can effectively prolong the survival of patients, it may increase the risk of bone marrow suppression and liver function abnormalities (21).

Regarding the clinical features observed, our research indicated that men experienced a more frequent occurrence of AEs with each of the quartet of tyrosine kinase inhibitors. In line with prior research, those patients over 65 years old exhibited a greater frequency of AEs after receiving therapies such as sunitinib, avapritinib, or ripretinib (22–24). As patients age, there is a deterioration in hepatic and renal function, culminating in diminished drug elimination and consequently, elevated concentrations of the medication in the bloodstream. This makes elderly patients with GISTs more prone to AEs during the use of TKIs. Compared with female patients, males have a larger body weight and body surface area, resulting in higher drug exposure and an increased risk of PPE and fatigue. At the same time, androgens affect hair follicle metabolism, which may increase the risk of hair loss and skin reactions. The implications of this research indicate that when it comes to prescribing imatinib and sunitinib, healthcare providers ought to give additional consideration to the male and elderly demographic.

Through our research, we found that the AEs caused by imatinib and sunitinib were mainly reported by healthcare professionals, including doctors, pharmacists and registered nurses. Nonetheless, the AEs linked to avapritinib and ripretinib were primarily reported by individuals outside the healthcare profession. The reports of AEs of imatinib and sunitinib are mainly from healthcare professionals, due to their mature therapeutic drug monitoring system, laboratory-dependent AEs and long-term clinical management needs. While for avapritinib and ripretinib, the proportion of reports from non-professionals has increased because of their short time on the market, more subjective symptoms and the increase in scenarios of patient self-management.

Within our research, we noted substantial variation in the typical initial occurrence time for AEs triggered by various pharmaceuticals. The median onset time for imatinib was as long as 251 days, while that for sunitinib was 70 days. The median onset times for ripretinib and avapritinib were similar, at 101 days and 98 days, respectively. Additionally, among the four TKI drugs, AEs were concentrated within the first month or after one year of administration. The results imply that surveillance for negative reactions ought to be tailored according to the specific medication involved. Moreover, we also evaluated the impact of AEs caused by different TKIs on mortality. Among the outcomes of these AEs, imatinib had the highest mortality rate at 59.9%, while avapritinib had the lowest at 8.3%. The high mortality rate of imatinib might be due to its most extensive use in GISTs, and the mortality might be caused by tumor progression rather than drug injury. In previous studies, the AEs of imatinib were usually controllable, and fatal AEs were rare (25, 26). However, in clinical trials, direct mortality data were limited, and most studies did not report treatment-related deaths (27). Therefore, our study further provides real-world data on imatinib-induced AEs mortality to optimize the use of imatinib in patients with GISTs.

Our study firstly conduct a data mining analysis on TKIs used for GISTs treatment based on FAERS database. Through the analysis toward AEs related with these TKIs, we draw a distribution map of AEs correlated with specific type of TKIs, which provided clinical guide when certain type of TKIs is conducted. We also revealed the outcome led by TKIs in GISTs treatment and the time-to-onset pattern of TKIs.

However, there are some shortcomings in this study. Since all data are acquired from FAERS database, there is lack of more detailed information. The data simply included basic information such as age, gender, weight, and medication but the risk degree of GISTs, whether the surgery was conducted is lack of description. Beside that, GISTs have higher incidence in Asian or Pacific island while the data in FAERS mostly comes from USA, which may led to reporting bias (1, 28) Moreover, Although the ROR quantifies the association between drugs and AEs, it cannot confirm causality due to limitations such as confounding factors, reporting biases, and absence of temporal data in spontaneous reports.

## 5 Conclusion

Since the use of imatinib in GISTs treatment, several type of TKIs are involved. Since the targets of different TKIs vary, the pattern of AEs led by certain type of TKIs varies. We conclude that sunitinib was highly correlated with cardiovascular disorders and patients with sunitinib should be closed monitored in cardiovascular function. While the cognitive function should be monitored when avapritinib is used. The AEs led by ripretinib tend to be slight compared with others and imatinib is highly correlated with death that in need of further follow-up.

## Data Availability

The original contributions presented in the study are included in the article/**Supplementary Material**. Further inquiries can be directed to the corresponding author.
